# Impact of route of access and stenosis subtype on outcome after transcatheter aortic valve replacement

**DOI:** 10.3389/fcvm.2023.1256112

**Published:** 2023-11-09

**Authors:** Julian Maier, Thomas Lambert, Thomas Senoner, Stephan Dobner, Uta Caroline Hoppe, Alexander Fellner, Bernhard Erich Pfeifer, Gudrun Maria Feuchtner, Guy Friedrich, Severin Semsroth, Nikolaos Bonaros, Johannes Holfeld, Silvana Müller, Markus Reinthaler, Clemens Steinwender, Fabian Barbieri

**Affiliations:** ^1^ Department of Cardiology, Kepler University Hospital, Linz, Austria; ^2^ Johannes Kepler University Linz, Medical Faculty, Linz, Austria; ^3^ Institute for Cardiovascular and Metabolic Research (ICMR), Johannes Kepler University Linz, Linz, Austria; ^4^ Institute of Pharmacology, Center for Physiology and Pharmacology, Medical University of Vienna, Vienna, Austria; ^5^ University Clinic of Internal Medicine III, Medical University Innsbruck, Innsbruck, Austria; ^6^ Department of Cardiology, Inselspital, Bern University Hospital, University of Bern, Bern, Switzerland; ^7^ 3rd Medical Department of Cardiology and Intensive Care Medicine, Clinic Ottakring (former Wilhelminenhospital), Vienna, Austria; ^8^ University Clinic of Internal Medicine II, Paracelsus Medical University, Salzburg, Austria; ^9^ Institute of Clinical Epidemiology, Tirol Kliniken, Innsbruck, Austria; ^10^ Division of Digital Medicine and Telehealth, University for Health Sciences, Medical Informatics and Technology (UMIT), Hall in Tirol, Austria; ^11^ Department of Radiology, Medical University Innsbruck, Innsbruck, Austria; ^12^ Department of Cardiac Surgery, Medical University Innsbruck, Innsbruck, Austria; ^13^ Department of Cardiology, Campus Benjamin Franklin, Charité–Universitätsmedizin Berlin, Corporate Member of Freie Universität Berlin, Humboldt–Universität zu Berlin, and Berlin Institute of Health, Berlin, Germany; ^14^ Institute of Active Polymers and Berlin-Brandenburg Center for Regenerative Therapies, Helmholtz-Zentrum Hereon, Teltow, Germany

**Keywords:** low-flow low-gradient aortic stenosis, transcatheter aortic valve implantation, transcatheter aortic valve replacement, access route, transapical access, transfemoral access, high gradient aortic stenosis

## Abstract

**Introduction:**

Previous analyses have reported the outcomes of transcatheter aortic valve replacement (TAVR) for patients with low-flow, low-gradient (LFLG) aortic stenosis (AS), without stratifying according to the route of access. Differences in mortality rates among access routes have been established for high-gradient (HG) patients and hypothesized to be even more pronounced in LFLG AS patients. This study aims to compare the outcomes of patients with LFLG or HG AS following transfemoral (TF) or transapical (TA) TAVR.

**Methods:**

A total of 910 patients, who underwent either TF or TA TAVR with a median follow-up of 2.22 (IQR: 1.22–4.03) years, were included in this multicenter cohort study. In total, 146 patients (16.04%) suffered from LFLG AS. The patients with HG and LFLG AS were stratified according to the route of access and compared statistically.

**Results:**

The operative mortality rates of patients with HG and LFLG were found to be comparable following TF access. The operative mortality rate was significantly increased for patients who underwent TA access [odds ratio (OR): 2.91 (1.54–5.48), *p* = 0.001] and patients with LFLG AS [OR: 2.27 (1.13–4.56), *p* = 0.02], which could be corroborated in a propensity score-matched subanalysis. The observed increase in the risk of operative mortality demonstrated an additive effect [OR for TA LFLG: 5.45 (2.35–12.62), *p* < 0.001]. LFLG patients who underwent TA access had significantly higher operative mortality rates (17.78%) compared with TF LFLG (3.96%, *p* = 0.016) and TA HG patients (6.36%, *p* = 0.024).

**Conclusions:**

HG patients experienced a twofold increase in operative mortality rates following TA compared with TF access, while LFLG patients had a fivefold increase in operative mortality rates. TA TAVR appears suboptimal for patients with LFLG AS. Prospective studies should be conducted to evaluate alternative options in cases where TF is not possible.

## Introduction

Aortic stenosis (AS) is the leading cause of transcatheter or surgical valvular interventions in North America and Europe (1). Current guidelines recommend transcatheter aortic valve replacement (TAVR) for patients with symptomatic severe AS and intermediate or high operative risk (1, 2). Several recent trials have demonstrated TAVR's non-inferiority compared with surgical aortic valve replacement (SAVR) in younger and lower risk populations (3). Due to its inherent minimal invasiveness, paucity of severe periprocedural complications, and shortened associated hospital stays, transfemoral (TF) access is considered the most favorable approach (1). Nevertheless, approximately 20% of planned TAVR procedures require alternative access routes, mainly due to extensive vessel calcification, tortuosity, or previously conducted vascular procedures (4). The utilization of transapical (TA) access, despite its higher rates of short- and midterm mortality, is still one of the most frequently employed alternatives (4–6). Although some long-term studies have provided comparisons of TF and TA TAVR clinical outcomes (5, 7), only few studies have focused on the subgroup of patients with low-flow, low-gradient (LFLG) AS patients undergoing TAVR. While TAVR in LFLG AS patients is associated with increased rates of early and midterm mortality (8–12), it is currently unknown how the selection of TF or TA access affects the clinical outcomes for these patients. We analyzed the data obtained from a non-randomized, multicenter study, comparing outcomes of patients with LFLG and high-gradient (HG) severe AS undergoing TF or TA TAVR with a median follow-up of 2.22 years and a long-term follow-up of up to 8 years.

## Methods

This multicenter analysis included data from patients who had treatment at the Medical University Innsbruck, Kepler University Hospital Linz, and Paracelsus Medical University Salzburg, Austria. The analysis included patients who underwent either TF or TA TAVR at the respective institutions between 2007 and 2017 (Supplementary Figure S1). The exclusion criteria were previous aortic valve interventions including valvuloplasty and subvalvular or non-severe aortic stenosis. In addition, patients with unknown coronary anatomy, previous endocarditis, recent acute coronary syndrome or resuscitation within the 2 months prior to intervention, conversion to SAVR, or another valvular defect classified as dominant disease were excluded in the study (13). Per guidelines, LFLG severe aortic stenosis is characterized by an aortic valve area of <1 cm^2^, a mean pressure gradient of <40 mmHg, and a stroke volume index (SVI) of ≤35 ml/m^2^. On the other hand, HG severe AS is defined by an aortic valve area of <1 cm^2^ and a mean pressure gradient of ≥40 mmHg (1). SVI was not routinely assessed in HG patients and is thus only presented for LFLG patients. The STS risk scores were calculated using the STS online risk calculator (https://riskcalc.sts.org/stswebriskcalc/calculate). Operative, all-cause, and cardiovascular (CV) mortality rates were considered endpoints. Operative mortality is defined as the occurrence of death during the index hospital stay up to 30 days following the intervention. All-cause and cardiovascular mortality data were obtained from Statistics Austria, the Federal Statistical Office of Austria. The study was approved by the local ethics committees and registered on https://clinicaltrials.gov/ (NCT02448485).

Categorical variables are reported as frequencies and percentages and compared using Pearson's chi-square test. Depending on the graphically assessed distribution, continuous variables are presented as mean values (±SD) or medians (IQR). Comparisons were conducted with the non-parametric Mann–Whitney *U*-test. To assess the differences between more than two groups, pairwise Wilcoxon rank sum tests with false discovery rate adjustment (Benjamini, Hochberg, and Yekutieli) were conducted. For survival analyses, the log-rank test was performed to test for the differences between groups. Kaplan–Meier curves were used for visualization. Univariate Cox, binary logistic, and binominal regression analyses were utilized to determine variables that should be included in the multiple regression analysis. Derived hazard ratios (HR) or odds ratios (OR) are given with a 95% confidence interval (CI). To minimize potential confounding factors, a propensity score matching model was calculated. Nearest neighbor matching was employed in order to match patients based on age, sex, and STS risk score, resulting in satisfactory standardized mean differences (Supplementary Figure S2). In total, there were 191 pairs of patients undergoing either TF or TA TAVR (Supplementary Table S1). All statistical analyses were performed using R Studio version 1.2.5033 (R Studio Inc., Boston, MA, USA) and SPSS Statistics version 27.0 (IBM, Armonk, NY, USA). The R packages that were used in this study are as follows: haven, survminer, survival, ggplot2, MatchIt, and glm. A *p*-value of <0.05 was considered statistically significant.

## Results

**Figure 1:**
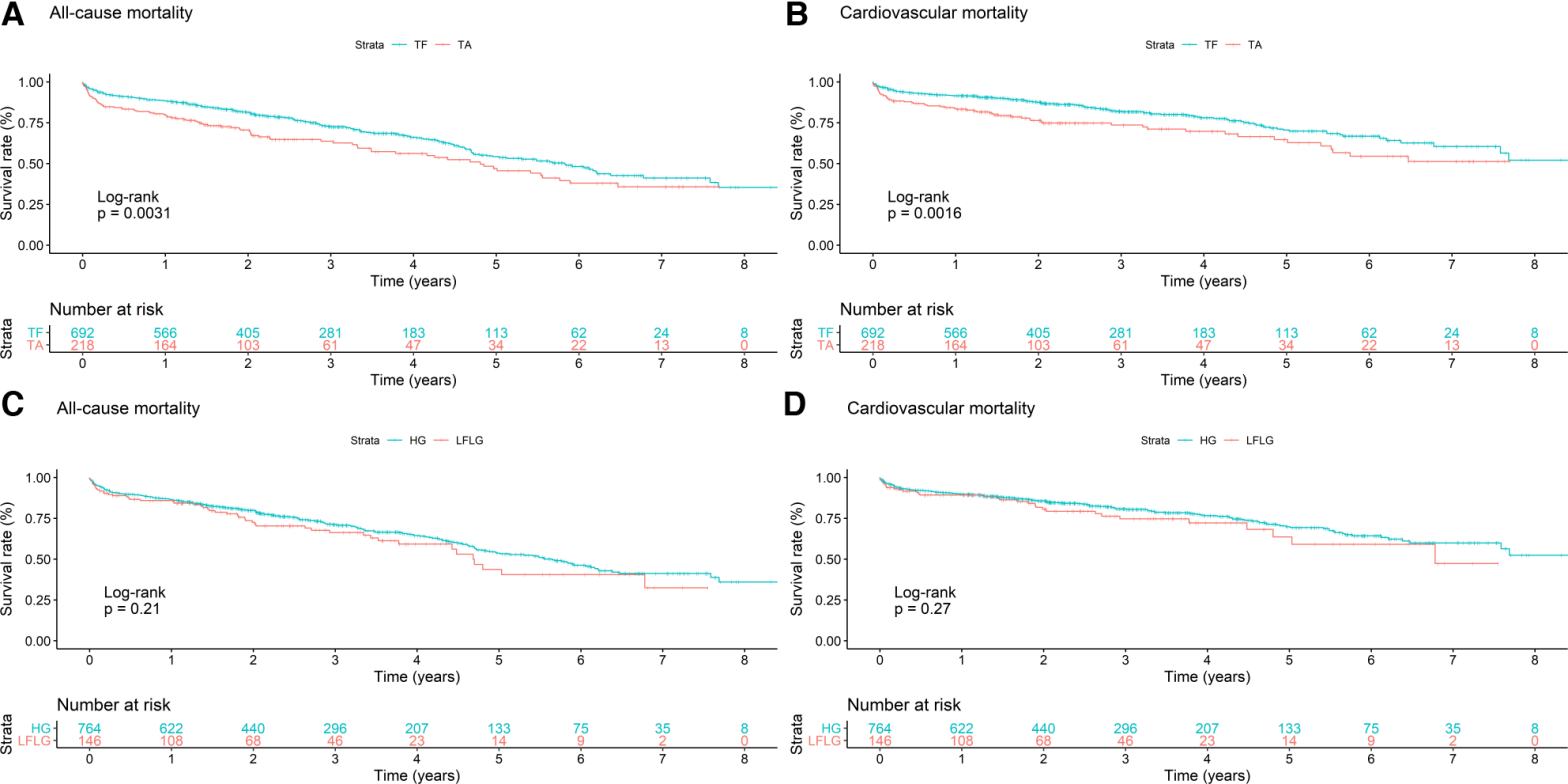
Kaplan–Meier curves with accompanying risk table of (**A**,**C**) all-cause and (**B**,**D**) cardiovascular mortality rates for patients (**A**,**B**) undergoing TF or TA TAVR or patients with (**C**,**D**) HG or LFLG AS.

In total, the study cohort included 910 patients of whom 692 (76.04%) underwent TF and 218 (23.96%) underwent TA TAVR (Table 1). A total of 764 patients (83.96%) suffered from HG and 146 (16.04%) patients suffered from LFLG severe AS (Table 2). After a median follow-up period of 2.22 years (1.22–4.03), the primary endpoint, which was all-cause mortality, was observed in 316 (34.73%) patients, with cardiovascular death being the most prevalent outcome (*n* = 197, 62.34%). In addition to complications occurring during the peri-interventional period (see Table 3), the results of the regression analyses revealed that both TA access (OR: 2.91; 95% CI: 1.54–5.48; *p* = 0.001) and LFLG AS (OR: 2.27; 95% CI: 1.13–4.56; *p* = 0.02) were significant predictors of operative mortality (Table 4). Operative mortality (OR: 2.91, 95% CI: 1.54–5.48, *p* = 0.001), as well as all-cause mortality (HR: 1.45, 95% CI: 1.13–1.85, *p* = 0.003) and CV mortality (HR: 1.62, 95% CI: 1.20–2.20, *p* = 0.002), was significantly higher in the TA cohort (Figures 1A,B). An increased mortality was mainly driven by elevated operative mortality and short-term mortality (Supplementary Figure S3). In LFLG patients, there was an observed increase in operative mortality, but not in all-cause or CV mortality (Figures 1C,D, Tables 1, 2, 3).

**Table 1:** Clinical characteristics and echocardiographic parameters of the study population subdivided into LFLG and HG groups for each route of access and compared statistically.

Variable	All (910)	TF (692, 76.04%)	TF LFLG (101)	TF HG (591)	*p*-value	TA (218, 23.96%)	TA LFLG (45)	TA HG (173)	*p*-value
Age	82.00 (78.00–85.00)	82.00 (79.00–85.00)	82.00 (77.00–85.00)	82.00 (79.00–85.00)	0.43	82.00 (76.00–84.00)	79.00 (75.00–83.50)	82.00 (76.00–84.00)	0.08
Female sex	494 (54.29%)	386 (55.78%)	42 (41.58%)	344 (58.21%)	**0**.**002**	108 (49.54%)	20 (44.44%)	88 (50.87%)	0.44
Body mass index (kg/m^2^)	25.83 (23.16–29.05)	25.80 (23.05–29.05)	25.33 (21.47–29.19)	25.88 (23.33–29.05)	0.08	25.92 (23.45–28.79)	25.26 (23.18–30.05)	26.17 (23.44–26.67)	0.77
STS risk of mortality (%)	3.66 (2.70–5.21)	3.50 (2.62–4.92)	3.68 (2.77–5.13)	3.48 (2.59–4.90)	0.34	4.50 (3.18–6.08)	4.76 (3.59–5.85)	4.32 (2.97–6.12)	0.571
Troponin T (pg/ml)	24.30 (16.00–40.22)	24.00 (16.00–39.70)	24.30 (16.88–40.08)	24.00 (16.00–39.35)	0.57	26.00 (16.20–45.30)	31.30 (17.55–51.05)	25.00 (16.00–43.48)	0.16
NT pro-BNP (ng/L)	2,338.50 (1,039.50–5,076.25)	2,355.00 (971.50–4,777.00)	2,910.00 (1,123.50–5,202.50)	2,255.50 (960.50–4,676.00)	0.149	2,657.00 (1,156.50–5,610.00)	3,584.00 (1,653.00–6,619.75)	2,243.00 (1,098.00–5,316.50)	0.06
eGFR (ml/min/1.73 m^2^)	70.42 (49.85–81.87)	70.91 (51.91–81.58)	68.71 (46.34–82.60)	71.27 (52:45–81:58)	0.549	67.20 (43.01–82.96)	58.27 (35.24–85.59)	67.59 (47.49–81.86)	0.566
Coronary artery disease									
None	204 (22.40%)	175 (25.29%)	22 (21.78%)	153 (25.89%)	0.38	29 (13.30%)	1 (2.22%)	28 (16.18%)	**0**.**01**
Diffuse sclerosis	272 (29.90%)	196 (28.32%)	14 (13.86%)	182 (30.79%)	**<0**.**001**	76 (34.86%)	14 (31.11%)	62 (35.84%)	0.55
One vessel	192 (21.10%)	153 (22.11%)	27 (26.73%)	126 (21.32%)	0.23	39 (17.89%)	8 (17.78%)	31 (17.92%)	0.98
Two vessel	95 (10.40%)	72 (10.40%)	15 (14.85%)	57 (9.65%)	0.11	23 (10.55%)	7 (15.556%)	16 (9.25%)	0.22
Three vessel	109 (12.00%)	70 (10.12%)	19 (18.82%)	51 (8.63%)	**0**.**002**	39 (17.89%)	12 (26.67%)	27 (15.61%)	0.085
Left main	38 (4.20%)	26 (3.76%)	4 (3.96%)	22 (3.72%)	0.91	12 (5.51%)	3 (6.67%)	9 (5.20%)	0.70
Arterial hypertension	761 (83.62%)	576 (83.24%)	84 (83.17%)	492 (83.25%)	0.90	185 (84.86%)	39 (86.67%)	146 (84.39%)	0.71
Diabetes mellitus II	234 (25.71%)	166 (23.99%)	26 (25.74%)	140 (23.69%)	0.66	68 (31.19%)	19 (42.22%)	49 (28.32%)	0.07
Hypercholesterinemia	492 (54.07%)	369 (53.32%)	66 (65.35%)	303 (51.27%)	**0**.**01**	123 (56.42%)	25 (55.56%)	98 (56.65%)	0.90
Chronic obstructive pulmonary disease	169 (18.57%)	115 (16.62%)	22 (21.78%)	93 (15.74%)	0.09	54 (24.77%)	9 (20.00%)	45 (26.01%)	0.41
Atrial fibrillation									
None	580 (63.74%)	450 (65.03%)	56 (55.45%)	394 (66.67%)	**0**.**03**	130 (59.63%)	22 (48.89%)	108 (62.43%)	0.10
Paroxysmal	128 (14.06%)	94 (13.58%)	13 (12.87%)	81 (13.70%)	0.82	34 (15.60%)	10 (22.22%)	24 (13.87%)	0.17
Non-paroxysmal	202 (22.20%)	148 (21.39%)	32 (31.68%)	116 (19.63%)	**0**.**007**	54 (24.77%)	13 (28.89%)	41 (23.70%)	0.47
Previous cerebrovascular accident	113 (12.42%)	47 (6.79%)	16 (15.84%)	58 (9.81%)	0.08	39 (17.89%)	9 (20.00%)	30 (17.34%)	0.68
Follow-up (years)	2.22 (1.22–4.03)	2.44 (1.27–4.19)	2.03 (1.05–3.78)	2.49 (1.33–4.30)	0.14	1.82 (1.00–3.49)	1.46 (0.38–2.37)	2.02 (1.07–4.02)	**0**.**02**
Cardiovascular mortality	197 (21.65%)	136 (19.65%)	17 (16.83%)	119 (20.14%)	0.44	61 (27.98%)	15 (33.33%)	46 (26.59%)	0.37
All-cause mortality	316 (34.73%)	226 (32.66%)	29 (28.71%)	197 (33.33%)	0.36	90 (41.28%)	21 (46.67%)	69 (39.88%)	0.41
Left ventricular ejection fraction (%)	55.00 (45.00–61.00)	55.00 (45.00–61.00)	45.00 (35.00–56.00)	55.00 (50.00–62.00)	**<0**.**001**	55.00 (46.00–60.00)	42.00 (29.50–55.50)	56.00 (50.00–62.00)	**<0**.**001**
Stroke volume index (ml/m^2^)	—	—	27.00 (21.00–32.75)	—	—	—	28.00 (22.75–30.00)	—	—
Mean syst. Gradient (mmHg)	49.00 (40.00–60.00)	50.00 (41.00–60.00)	33.00 (27.00–36.00)	51.00 (45.00–60.00)	**<0**.**001**	46.00 (40.00–58.00)	32.00 (28.00–35.00)	50.00 (44.00–61.00)	**<0**.**001**
Max. syst. Gradient (mmHg)	80.00 (68.00–94.00)	80.00 (69.25–94.00)	55.00 (45.00–62.00)	83.00 (72.00–95.00)	**<0**.**001**	79.00 (64.00–94.00)	50.50 (44.25–59.00)	81.00 (72.00–95.00)	**<0**.**001**
Valve area (cm^2^)	0.60 (0.50–0.71)	0.60 (0.50–0.70)	0.65 (0.55–0.80)	0.60 (0.50–0.70)	**0**.**002**	0.63 (0.50–0.75)	0.70 (0.56–0.80)	0.60 (0.50–0.72)	**0**.**04**
Indexed valve area (Mosteller)	0.34 (0.28–0.40)	0.33 (0.27–0.39)	0.34 (0.29–0.43)	0.33 (0.27–0.39)	**0**.**015**	0.35 (0.29–0.41)	0.38 (0.31–0.42)	0.34 (0.29–0.41)	0.096
End-diastolic septum width (mm)	14.60 (13.00–17.00)	14.95 (13.00–16.63)	14.00 (12.60–16.00)	15.00 (13.00–17.00)	**0**.**028**	14.05 (12.70–17.55)	13.85 (12.33–15.00)	14.70 (12.70–18.00)	0.06
Mitral regurgitation									
0	165 (18.13%)	112 (16.18%)	6 (5.94%)	106 (17.94%)	**0**.**003**	53 (24.31%)	7 (15.56%)	46 (26.59%)	0.067
1	555 (60.99%)	421 (60.84%)	56 (55.45%)	365 (61.76%)	0.16	134 (61.47%)	27 (60.00%)	107 (61.85%)	0.87
2	175 (19.23%)	144 (20.81%)	35 (34.65%)	109 (18.44%)	**<0**.**001**	31 (14.22%)	11 (24.44%)	20 (11.56%)	**0**.**025**
3	15 (1.65%)	15 (2.17%)	4 (3.96%)	11 (1.86%)	0.19	—	—	—	
Aortic regurgitation									
0	355 (39.01%)	265 (38.30%)	37 (36.64%)	228 (38.58%)	0.85	90 (41.28%)	20 (44.44%)	70 (40.46%)	0.32
1	471 (51.76%)	362 (52.31%)	50 (49.50%)	312 (52.79%)	0.43	109 (50.00%)	23 (51.11%)	86 (49.71%)	0.83
2	82 (9.01%)	63 (9.10%)	14 (13.86%)	49 (8.29%)	0.08	19 (8.72%)	2 (4.44%)	17 (9.83%)	0.22
3	2 (0.22%)	2 (0.29%)	—	2 (0.34%)	0.56	—	—	—	

Bold indicates *p*-values < 0.05.

**Table 2:** Clinical characteristics and echocardiographic parameters of the study population subdivided into TF and TA groups for each AS subtype and compared statistically.

Variable	All (910)	HG (764, 83.96%)	TF HG (591)	TA HG (173)	*p*-value	LFLG (146, 16.04%)	TF LFLG (101)	TA LFLG (45)	*p*-value
Age	82.00 (78.00–85.00)	82.00 (79.00–85.00)	82.00 (79.00–85.00)	82.00 (76.00–84.00)	**0**.**01**	82.00 (77.00–85.00)	82.00 (77.00–85.00)	79.00 (75.00–83.50)	**0**.**01**
Female sex	494 (54.29%)	432 (56.54%)	344 (58.21%)	88 (50.87%)	0.09	62 (42.47%)	42 (41.58%)	20 (44.44%)	0.75
Body mass index (kg/m^2^)	25.83 (23.16–29.05)	25.91 (23.38–28.89)	25.88 (23.33–29.05)	26.17 (23.44–26.67)	0.71	25.31 (22.09–29.30)	25.33 (21.47–29.19)	25.26 (23.18–30.05)	0.35
STS risk of mortality (%)	3.66 (2.70–5.21)	3.62 (2.67–5.20)	3.48 (2.59–4.90)	4.32 (2.97–6.12)	**<0**.**001**	3.83 (2.90–5.41)	3.68 (2.77–5.13)	4.76 (3.59–5.85)	**0**.**02**
Troponin T (pg/ml)	24.30 (16.00–40.22)	24.00 (16.00–40.00)	24.00 (16.00–39.35)	25.00 (16.00–43.48)	0.50	26.70 (17.00–43.90)	24.30 (16.88–40.08)	31.30 (17.55–51.05)	0.20
NT pro-BNP (ng/L)	2,338.50 (1,039.50–5,076.25)	2,243.00 (1,009.00–4,784.00)	2,255.50 (960.50–4,676.00)	2,243.00 (1,098.00–5,316.50)	0.43	3,022.00 (1,514.50–5,408.00)	2,910.00 (1,123.50–5,202.50)	3,584.00 (1,653.00–6,619.75)	0.19
eGFR (ml/min/1.73 m^2^)	70.42 (49.85–81.87)	70.66 (51.37–81.58)	71.27 (52:45–81:58)	67.59 (47.49–81.86)	0.22	67.69 (43.62–83.08)	68.71 (46.34–82.60)	58.27 (35.24–85.59)	0.38
Coronary artery disease									
None	204 (22.40%)	181 (23.69%)	153 (25.89%)	28 (16.18%)	**0.01**	23 (15.75%)	22 (21.78%)	1 (2.22%)	**<0**.**01**
Diffuse sclerosis	272 (29.90%)	244 (31.94%)	182 (30.79%)	62 (35.84%)	0.21	28 (19.18%)	14 (13.86%)	14 (31.11%)	**0**.**02**
One vessel	192 (21.10%)	157 (20.55%)	126 (21.32%)	31 (17.92%)	0.33	35 (23.97%)	27 (26.73%)	8 (17.78%)	0.24
Two vessel	95 (10.40%)	73 (9.55%)	57 (9.65%)	16 (9.25%)	0.88	22 (15.07%)	15 (14.85%)	7 (15.556%)	0.91
Three vessel	109 (12.00%)	78 (10.21%)	51 (8.63%)	27 (15.61%)	**0.01**	31 (21.23%)	19 (18.82%)	12 (26.67%)	0.28
Left main	38 (4.20%)	31 (4.06%)	22 (3.72%)	9 (5.20%)	0.39	7 (4.79%)	4 (3.96%)	3 (6.67%)	0.48
Arterial hypertension	761 (83.62%)	638 (83.51%)	492 (83.25%)	146 (84.39%)	0.82	123 (84.25%)	84 (83.17%)	39 (86.67%)	0.59
Diabetes mellitus II	234 (25.71%)	189 (24.74%)	140 (23.69%)	49 (28.32%)	0.22	45 (30.82%)	26 (25.74%)	19 (42.22%)	**0.04**
Hypercholesterinemia	492 (54.07%)	401 (52.49%)	303 (51.27%)	98 (56.65%)	0.23	91 (62.33%)	66 (65.35%)	25 (55.56%)	0.26
Chronic obstructive pulmonary disease	169 (18.57%)	138 (18.06%)	93 (15.74%)	45 (26.01%)	**<0**.**01**	31 (21.23%)	22 (21.78%)	9 (20.00%)	0.79
Atrial fibrillation									
None	580 (63.74%)	502 (65.71%)	394 (66.67%)	108 (62.43%)	0.31	78 (53.42%)	56 (55.45%)	22 (48.89%)	0.46
Paroxysmal	128 (14.06%)	105 (13.74%)	81 (13.70%)	24 (13.87%)	0.96	23 (15.75%)	13 (12.87%)	10 (22.22%)	0.15
Non-paroxysmal	202 (22.20%)	157 (20.55%)	116 (19.63%)	41 (23.70%)	0.25	45 (30.82%)	32 (31.68%)	13 (28.89%)	0.74
Previous cerebrovascular accident	113 (12.42%)	88 (11.52%)	58 (9.81%)	30 (17.34%)	**<0**.**01**	25 (17.12%)	16 (15.84%)	9 (20.00%)	0.54
Follow-up (years)	2.22 (1.22–4.03)	2.28 (1.27–4.26)	2.49 (1.33–4.30)	2.02 (1.07–4.02)	**0**.**04**	1.82 (0.77–3.54)	2.03 (1.05–3.78)	1.46 (0.38–2.37)	**0**.**01**
Cardiovascular mortality	197 (21.65%)	165 (21.60%)	119 (20.14%)	46 (26.59%)	0.07	32 (21.92%)	17 (16.83%)	15 (33.33%)	**0**.**02**
All-cause mortality	316 (34.73%)	266 (34.82%)	197 (33.33%)	69 (39.88%)	0.11	50 (34.25%)	29 (28.71%)	21 (46.67%)	**0**.**03**
Left ventricular ejection fraction (%)	55.00 (45.00–61.00)	56.00 (50.00–62.00)	55.00 (50.00–62.00)	56.00 (50.00–62.00)	0.61	45.00 (33.00–55.25)	45.00 (35.00–56.00)	42.00 (29.50–55.50)	0.29
Mean syst. gradient (mmHg)	49.00 (40.00–60.00)	51.00 (45.00–60.00)	51.00 (45.00–60.00)	50.00 (44.00–61.00)	0.32	32.00 (27.00–36.00)	33.00 (27.00–36.00)	0.87 ± 5.78 @ 32.00 (28.00–35.00)	0.60
Max. syst. Gradient (mmHg)	80.00 (68.00–94.00)	83.00 (72.00–95.00)	83.00 (72.00–95.00)	81.00 (72.00–95.00)	0.87	53.00 (45.00–60.00)	55.00 (45.00–62.00)	50.50 (44.25–59.00)	0.13
Valve area (cm^2^)	0.60 (0.50–0.71)	0.60 (0.50–0.70)	0.60 (0.50–0.70)	0.60 (0.50–0.72)	0.14	0.67 (0.55–0.80)	0.65 (0.55–0.80)	0.70 (0.56–0.80)	0.33
Indexed valve area (Mosteller)	0.34 (0.28–0.40)	0.33 (0.27–0.39)	0.33 (0.27–0.39)	0.34 (0.29–0.41)	0.84	0.36 (0.30–0.42)	0.34 (0.29–0.43)	0.38 (0.31–0.42)	0.47
End-diastolic septum width (mm)	14.60 (13.00–17.00)	15.00 (13.00–17.00)	15.00 (13.00–17.00)	14.70 (12.70–18.00)	0.45	14.00 (12.60–15.85)	14.00 (12.60–16.00)	13.85 (12.33–15.00)	0.62
Mitral regurgitation									
0	165 (18.13%)	151 (19.76%)	106 (17.94%)	46 (26.59%)	**0**.**03**	13 (8.90%)	6 (5.94%)56	7 (15.56%)	0.20
1	555 (60.99%)	472 (61.78%)	365 (61.76%)	107 (61.85%)	0.71	83 (56.85%)	(55.45%)	27 (60.00%)	0.38
2	175 (19.23%)	129 (16.88%)	109 (18.44%)	20 (11.56%)	**0**.**04**	46 (31.51%)	35 (34.65%)	11 (24.44%)	0.29
3	15 (1.65%)	11 (1.44%)	11 (1.86%)	—	0.07	4 (2.74%)	4 (3.96%)	—	0.19
Aortic regurgitation									
0	355 (39.01%)	298 (39.01%)	228 (38.58%)	70 (40.46%)	0.63	57 (39.04%)	37 (36.64%)	20 (44.44%)	0.42
1	471 (51.76%)	398 (52.09%)	312 (52.79%)	86 (49.71%)	0.97	73 (50.00%)	50 (49.50%)	23 (51.11%)	0.77
2	82 (9.01%)	66 (8.64%)	49 (8.29%)	17 (9.83%)	0.38	16 (10.96%)	14 (13.86%)	2 (4.44%)	0.10
3	2 (0.22%)	2 (0.26%)	2 (0.34%)	—	0.46	8 (5.48%)	—	—	—

Bold indicates *p*-values < 0.05.

**Table 3:** Procedural characteristics of the study population subdivided into LFLG and HG groups for each route of access and compared statistically.

Variable	All (910)	TF (692, 76.04%)	TF LFLG (101)	TF HG (591)	*p*-value	TA (218, 23.96%)	TA LFLG (45)	TA HG (173)	*p*-value
Prosthesis type									
Sapien	514 (56.48%)	300 (43.35%)	51 (50.50%)	248 (41.96%)	0.089	214 (98.16%)	45 (100%)	169 (97.69%)	0.37
CoreValve	370 (40.66%)	369 (53.32%)	48 (47.53%)	321 (54.32%)	0.17	1 (0.46%)		1 (0.58%)	0.61
Other	26 (2.86%)	23 (3.33%)	1 (0.99%)	22 (3.72%)	0.30	3 (1.38%)		3 (1.73%)	0.47
Valve size	26.00 (26.00–29.00)	29.00 (26.00–29.00)	29.00 (26.00–29.00)	29.00 (26.00–29.00)	0.99	26.00 (23.00–26.00)	26.00 (23.00–26.00)	26.00 (23.00–26.00)	0.96
Dressler syndrome	6 (0.66%)	1 (0.15%)	—	1 (0.17%)	0.68	5 (2.29%)	1 (2.22%)	4 (2.31%)	0.92
Pericardiocentesis	17 (1.87%)	13 (1.88%)	—	13 (2.20%)	0.13	4 (1.83%)	–	4 (2.31%)	0.30
Thoracentesis	86 (9.45%)	29 (4.19%)	6 (5.94%)	23 (3.89%)	0.44	57 (26.15%)	12 (26.67%)	45 (26.01%)	0.92
Operative mortality	41 (4.51%)	22 (3.12%)	4 (3.96%)	18 (3.05%)	0.63	19 (8.72%)	8 (17.78%)	11 (6.36%)	**0**.**016**
Renal failure	149 (16.37%)	105 (15.17%)	11 (10.89%)	94 (15.91%)	0.19	44 (20.18%)	13 (28.89%)	31 (17.92%)	0.087
Periprocedural complications necessitating redo	60 (6.59%)	38 (5.49%)	7 (6.93%)	31 (5.25%)	0.50	22 (10.09%)	3 (6.67%)	19 (10.98%)	0.39
Cerebrovascular accident	22 (2.42%)	19 (2.75%)	5 (4.95%)	14 (2.37%)	0.17	3 (1.38%)	5 (11.11%)	3 (1.73%)	0.36
Mechanical ventilation >24 h	36 (3.96%)	19 (2.75%)	3 (2.97%)	16 (2.71%)	0.94	17 (7.80%)	5 (11.11%)	12 (6.94%)	0.43
Mean syst. gradient (mmHg)	9.00 (7.00–13.00)	9.00 (6.00–12.00)	8.00 (7.00–10.00)	9.00 (6.00–12.00)	0.1	11.00 (9.00–15.00)	10.00 (8.00–14.50)	11.50 (9.00–15.00)	0.49
Max. syst. gradient (mmHg)	17.00 (12.00–17.00)	16.00 (12.00–21.00)	15.00 (12.50–18.00)	16.00 (12.00–22.00)	0.28	20.00 (15.00–26.00)	18.00 (15.00–27.50)	20.00 (15.00–26.00)	1.00
Paravalvular leak	529 (58.13%)	463 (66.91%)	65 (64.36%)	398 (67.34%)	0.17	66 (30.28%)	15 (33.33%)	51 (29.48%)	0.51

Bold indicates *p*-values < 0.05.

**Table 4:** Univariate and multiple Cox regression with CV mortality as response and univariate binary logistic regression with operative mortality as response.

	CV mortality						Operative mortality		
	Unadjusted hazard ratio	95% CI	*p*-value	Adjusted hazard ratio	95% CI	*p*-value	Unadjusted odds ratio	95% CI	*p*-value
Sex	0.82	0.62–1.09	0.17				0.93	0.52–1.82	0.93
Arterial hypertension	0.81	0.56–1.16	0.25				0.93	0.40–2.14	0.86
Diabetes mellitus II	1.29	0.96–1.76	0.11				1.06	0.52–2.15	0.87
NT pro-BNP	1.000023	1.000012–1.000035	**<0**.**001**	1.00	0.99–1.00	0.65	1.00	1.00–1.00	0.16
Troponin T	1.001	1.00–1.001	**0**.**012**	1.00	0.99–1.00	0.77	1.00	0.99–1.00	0.96
Creatinine	1.38	1.19–1.59	**<0**.**001**	0.90	0.56–1.43	0.65	1.33	1.00–1.77	**0**.**04**
Renal failure (post-operative)	2.10	1.52–2.90	**<0**.**001**	2.16	1.02–4.56	**0**.**04**	4.90	2.58–9.31	**<0**.**001**
Low-flow, low-gradient	1.24	0.85–1.81	0.29				2.27	1.13–4.56	**0**.**02**
Left ventricular ejection fraction (%)	0.98	0.97–0.99	**<0**.**001**	0.98	0.96–1.01	0.13	0.94	0.91–0.98	**0**.**003**
STS score	1.04	1.02–1.06	**<0**.**001**	1.04	0.94–1.16	0.43	1.12	1.01–1.24	**0**.**04**
Atrial fibrillation (pre-operative)	1.46	1.10–1.94	**0**.**009**	2.47	0.28–21.72	0.42	1.62	0.86–3.07	0.14
Post-operative atrial fibrillation	1.07	1.02–0.1.12	**0**.**005**	0.67	0.27–1.68	0.39	1.23	0.99–1.53	0.06
Procedure (transapical)	1.62	1.20–2.20	**0**.**002**	1.12	0.61–2.06	0.71	2.91	1.54–5.48	**0**.**001**
Periprocedural complications necessitating redo	1.47	0.92–2.36	0.11				3.80	1.67–8.65	**0**.**001**
Apoplex (peri-interventional)	2.33	1.23–4.41	**0**.**009**	0.69	0.07–5.70	0.69	10.89	4.16–28.51	**<0**.**001**
Mechanical ventilation >24h	4.75	2.98–7.57	**<0**.**001**	2.65	0.87–8.08	0.09	20.66	9.57–44.59	**<0**.**001**
Pericardiocentesis	3.06	1.61–5.93	**0**.**003**	3.73	1.38–10.10	**0**.**01**	15.91	5.20–48.91	**<0**.**001**
Thoracentesis	3.30	2.29–4.76	**<0**.**001**	3.26	1.65–6.43	**0**.**001**	6.73	3.12–14.55	**<0**.**001**

Bold indicates *p*-values < 0.05.

In the TF cohort (*n* = 692), 101 patients (4.59%) suffered from LFLG AS, and 591 (85.41%) patients presented with HG AS. TA access was chosen for 218 patients, 45 patients with LFLG (20.64%) and 173 (79.36%) patients with HG AS. While patients with LFLG AS were predominantly male, this difference was more pronounced in the TF group (Table 1). The R2-CHA2DS2-VAsc scores, but not STS risk scores, were significantly higher in the LFLG group for both access routes. CAD, particularly the three-vessel CAD, and concomitant mitral regurgitation were more common in patients with LFLG, irrespective of the chosen approach for TAVR. The majority of LFLG patients in both groups was found to have reduced LVEF [*n* = 87, 59.59%, LVEF: 35.00% (27.00–41.00); vs. patients with preserved LVEF: *n* = 59, 40.41%, LVEF: 59.00% (53.00–63.00)]. Periprocedural events were comparable for all groups (Table 3).

**Figure 2:**
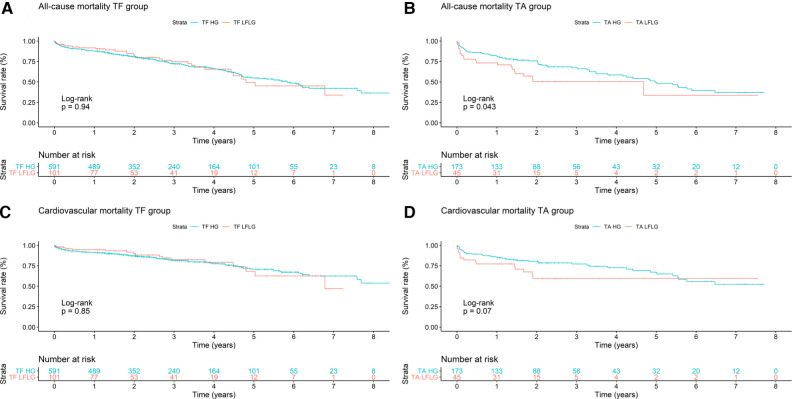
Kaplan–Meier curves with accompanying risk table of (**A**) TF all-cause, (**B**) TA all-cause, (**C**) TF cardiovascular, and (**D**) TA cardiovascular mortality rates for HG and LFLG severe aortic stenosis patients.

**Figure 3:**
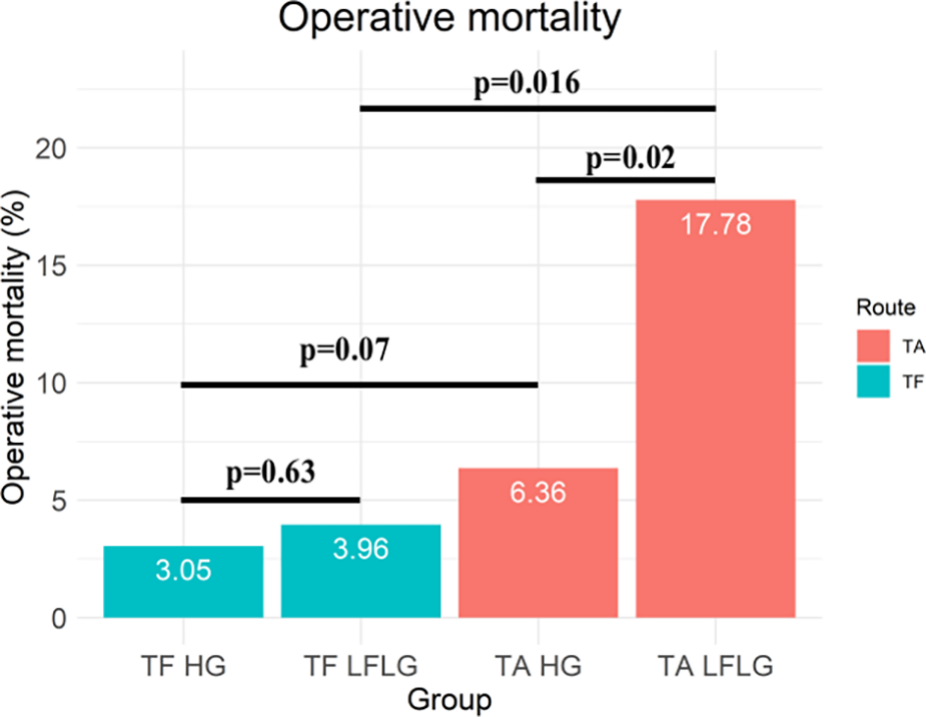
Central illustration. Operative mortality for patients with HG and LFLG severe aortic stenosis undergoing TF and TA TAVR.

After TF access, all-cause (*p* = 0.94) and CV (*p* = 0.85) mortality did not significantly differ between patients with LFLG or HG AS (Figure 2). In contrast, after TA access, all-cause mortality rate was significantly higher in LFLG patients (*p* = 0.04), whereas only a trend for increased CV mortality rates was noticeable (*p* = 0.07). The operative mortality rate after TA TAVR was significantly increased in the LFLG cohort (17.78%, *n* = 8) compared with the HG cohort (6.36%, *n* = 11, *p* = 0.02; OR for TA LFLG: 5.45; 95% CI: 2.35–12.62, *p* < 0.001, Figure 3–Central illustration). The operative mortality rate after TF TAVR did not differ between LFLG (3.96%, *n* = 4) and HG patients (3.05%, *n* = 18, *p* = 0.63, Figure 3—Central illustration, Supplementary Figure S4). In a binary logistic regression model, exploring the differences in the operative mortality rate among the four groups in more detail, only the group of TA LFLG patients differed significantly (*p* = 0.01) from the reference category TF LFLG with an OR of 5.24 (95% CI: 1.55–20.62).

**Figure 4:**
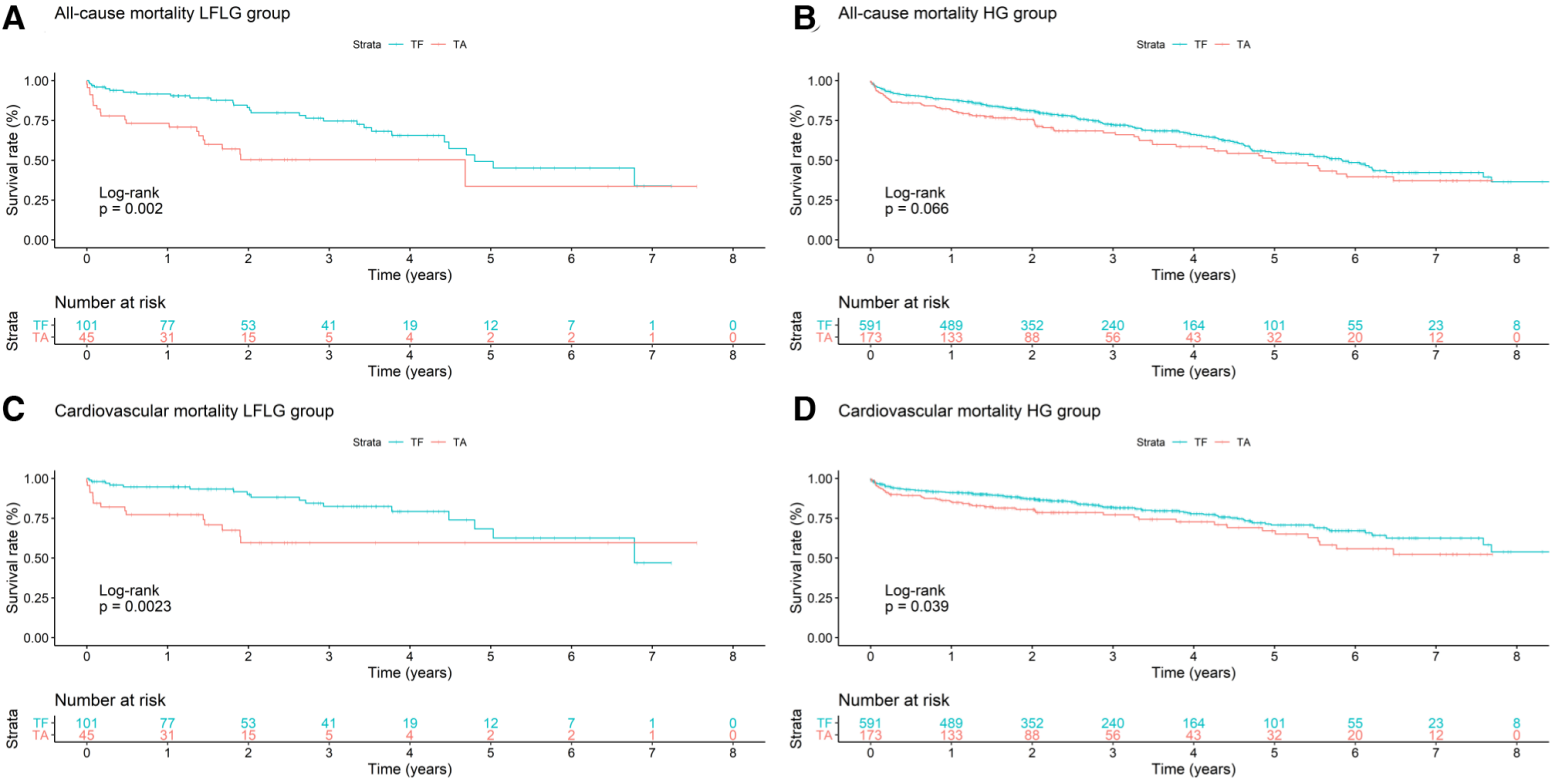
Kaplan–Meier curves with accompanying risk table of (**A**) all-cause and (**C**) cardiovascular mortality rates for LFLG severe aortic stenosis patients undergoing TF or TA TAVR and (**B**) all-cause and (**D**) cardiovascular mortality rates for HG severe aortic stenosis patients with respect to TF or TA routes of access.

For both HG and LFLG AS, TA patients exhibited higher STS risk scores and were slightly younger than their TF counterparts (Table 2). The selection of access route did not significantly affect the all-cause mortality rate in HG AS patients (Figure 4; HR: 1.29; 95% CI: 0.98–1.70; *p* = 0.07), while the CV mortality rate was increased after TA access (HR: 1.43; 95% CI: 1.02–2.01; *p* = 0.04). The operative mortality rate was twofold higher in HG patients undergoing TA, compared with that of the TF TAVR (6.36% vs. 3.05%; *n* = 11 vs. 18; *p* = 0.07). In LFLG patients, these effects were more pronounced. The CV (HR: 2.87; 95% CI: 1.41–5.83; *p* = 0.004) and all-cause mortality rates (HR: 2.40; 95% CI: 1.35–4.25; *p* = 0.003) were significantly higher in the TA cohort. The operative mortality rate was fivefold higher for LFLG patients after TA, compared with that of the TF TAVR (17.78% vs. 3.96%, *n* = 8 vs. *n* = 4, *p* = 0.016). In a propensity score-matched subanalysis, which also adjusted for STS risk scores, the operative mortality rate remained significantly higher for patients suffering from LFLG AS (Supplementary Table S2). In LFLG patients, a reduced LVEF was not an indicator of operative mortality rate, but it was associated with increased all-cause and CV mortality rates (Supplementary Table S3). The strongest predictors of CV and operative mortality rates were various peri-interventional complications, which occurred more frequently after TA access (Tables 3, 4).

## Discussion

After more than a decade, TAVR has now been well established as the treatment of choice for intermediate and high-risk patients suffering from severe aortic stenosis (1). As the procedure is still evolving further, focus has now shifted to reducing TAVR-associated morbidity and mortality. In the early stages of TAVR, TF and TA were the preferred routes of access, while other alternative routes (e.g., transsubclavian, transaxillary, transaortic) were infrequently reported (14). The higher mortality rate associated with TA access has now strengthened TF access as the primary route of access, while alternatives to TA access, such as transcarotid, transsubclavian, or transaxillary access, have also been developed and are chosen based on individual factors, including the expertise of the center and operator (5, 15, 16).

In our non-randomized, multicenter cohort study, we investigated the selection between TF and TA access and their effects on mortality rates in the higher risk population suffering from LFLG aortic stenosis. The main and novel finding of our study suggests that the choice of access route is of higher importance in high-risk patient populations. It was previously described that there is an increased risk associated with TA access in an unselected TAVR population (5, 15). Interestingly, we found that clinical outcomes in LFLG and HG patients were comparable after TF access, yet TA access was found to be associated with a significant fivefold increase in operative mortality rate in LFLG AS patients compared with TF access. The mortality rate among HG AS patients was found to be twice as high following the TA access. The selection of access route is typically limited by the presence of patient co-morbidities, which was also apparent in our cohort. TA TAVR patients more often suffered from advanced cardiovascular disease, atrial fibrillation, and secondary mitral regurgitation. The elevated burden of co-morbidities was also reflected by the higher STS scores observed in patients undergoing TA TAVR.

Further subgroup analysis of LFLG patients demonstrated an increased risk of long-term CV and all-cause mortality rates for patients with reduced LVEF compared with those with preserved LVEF. While these data should be interpreted cautiously due to the small sample size, the regression analyses revealed higher LVEF to be a protective factor independent of AS subtype, in line with current understanding (17). The impact of CAD on TAVR outcomes is currently debated as well, with conflicting evidence being reported (18). While a previous study showed the presence of multivessel CAD to predict mortality rates in LFLG patients, our models did not imply that CAD was a significant predictor of worsened outcome (19). The findings concerning predictors of mortality rates after TAVR are not heterogenous (20), and both, individual co-morbidities and the occurrence of peri-interventional complications, which are more frequently associated with TA access, have been shown to influence patient outcomes (21–24).

In our stratified analysis, the previously reported increased mortality rate of LFLG patients compared with HG patients was only evident in cases where TA access was utilized, but not observed for patients with TF access (8, 25). When comparing outcomes following TA access between LFLG and HG patients, we noticed significant increases in the operative, CV, and all-cause mortality rates for LFLG patients. The described increase in the operative mortality rate for LFLG patients undergoing TA TAVR is likely attributable to a combination of factors that include: (i) the higher complication rates associated with TA access compared with TF TAVR, (ii) an increased occurrence of myocardial injury after TA TAVR, (iii) LFLG patients having more co-morbidities and a higher operative risk, and (iv) LFLG patients additionally exhibiting higher degrees of interstitial myocardial fibrosis and changes in myocardial wall thickness when compared with HG patients (26–28). In addition to established co-morbidities, a clustering of transthyretin (TTR) amyloidosis in LFLG patients (up to 30% prevalence) can now be reasonably assumed, which may further explain the adverse outcomes observed in comparison with HG AS patients. TTR amyloid deposition initiates at the base of the heart before progressing toward the apex. The presence of apical sparing with preserved apical contractility is frequently observed until the illness reaches its advanced stages (29). The selection of a TA approach for these patients results in myocardial injury targeting the most vulnerable area (30). In addition, our findings might be generalizable to other TA approaches, such as transapical mitral valve replacement, particularly in AS patients with concomitant mitral regurgitation (31, 32).

In HG AS patients with lower risk, TA TAVR was associated with a twofold increase in operative mortality rate. While this increase in mortality rate may be acceptable until evidence suggests a better alternative, an evaluation of access routes other than TA should be performed, before settling on the final route of access for LFLG AS patients. Rather than proceeding with TA TAVR in LFLG patients, if TF access is not feasible, a referral to an experienced center offering alternative access routes should be considered. Future studies need to address which alternative access routes are safest for high-risk patients, in whom TF access is not feasible.

This study is subjected to several limitations. While the multicenter design of this study has its advantages concerning the generalizability of results, one disadvantage is that echocardiograms were taken at each site, and a core facility analysis was not available. Due to the observational nature of our study, we are unable to exclude selection bias. We tried to counteract this by extending the period of data inclusion and by additionally conducting propensity score match-based analyses, corroborating major findings made for the whole cohort. A general problem with registries is the handling of missing data. We had to exclude patients with incomplete data sets, which might skew the results. Due to the rather small number of LFLG patients, further sub-analyses, e.g., of preserved or reduced LVEF LFLG patients, were abridged. Nevertheless, our results corroborate previous meta-analyses. Ethically, it seems debatable whether future prospective randomized trials comparing outcomes of alternative routes of access in this population should even include TA access due to the herein reported increased risk of operative mortality rates. We are unable to exclude that confounders influencing outcome in LFLG patients were missed by us. Future studies are therefore necessary to enable more precise risk stratification within the LFLG population.

In conclusion, while TF TAVR is safe, even in high-risk patient populations suffering from LFLG AS, TA access is associated with increased mortality after TAVR. With a fivefold increase in the operative mortality rate, LFLG AS patients are at a particularly high risk. Prospective studies should be conducted to evaluate alternative options in cases where TF is not feasible.

## Data Availability

The raw data supporting the conclusions of this article will be made available by the authors, upon reasonable request.

## In Memoriam

In memoriam of Christoph Sautner MD, his valuable contributions and camaraderie.
