# Supplementary material for: Impact of route of access and stenosis subtype on outcome after transcatheter aortic valve replacement
Source: Front Cardiovasc Med. 2023 Nov 9;10:1256112. doi: 10.3389/fcvm.2023.1256112 (PMC10665844; doi:10.3389/fcvm.2023.1256112)
Supplement: Supplementary file 1 [file Datasheet1.docx]

**Supplemental Material**

**Supplemental Figures**

**Supplemental Tables**

**Supplemental Figures**

**
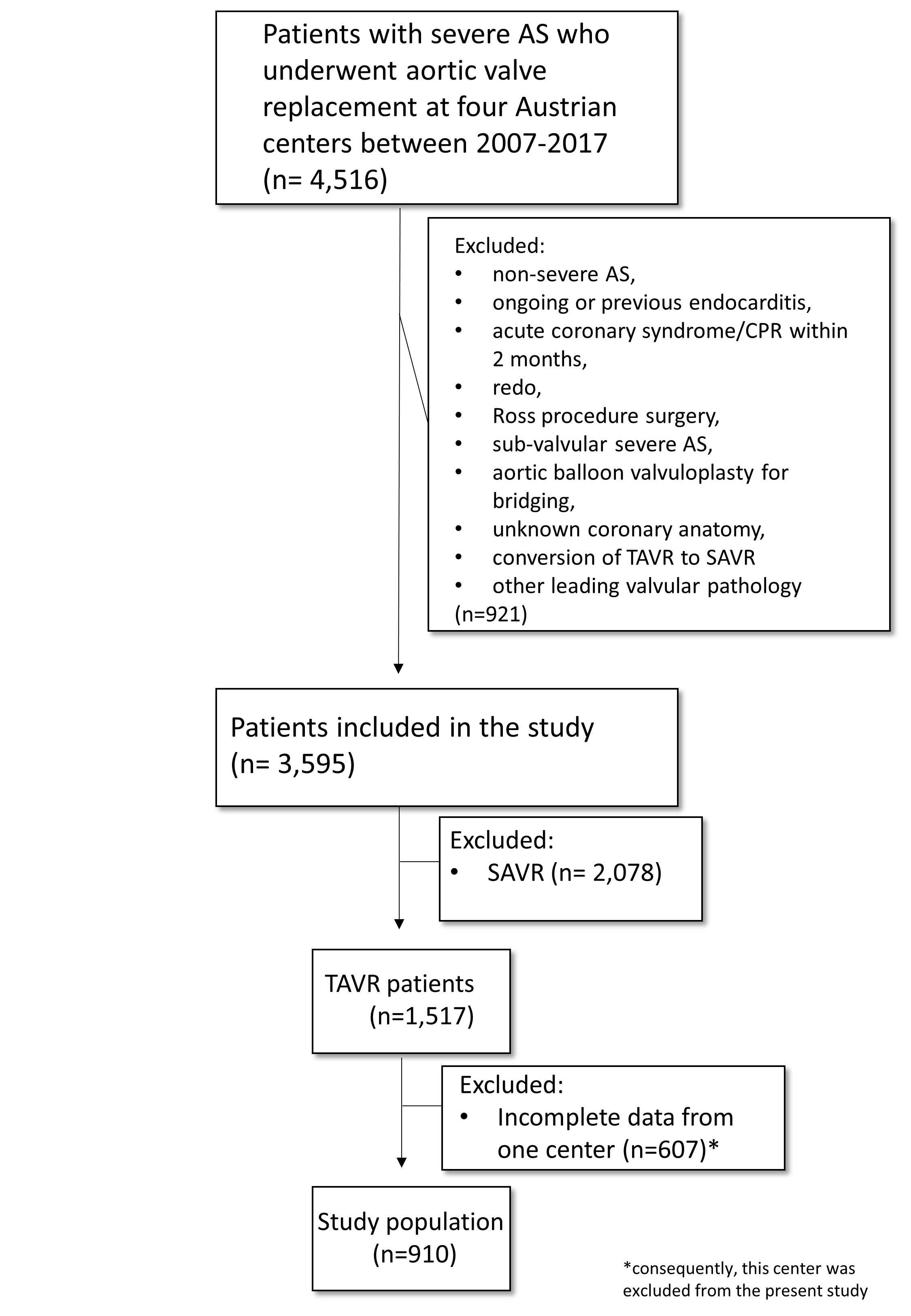
**

Supplemental Figure 1: Study flowchart.

**
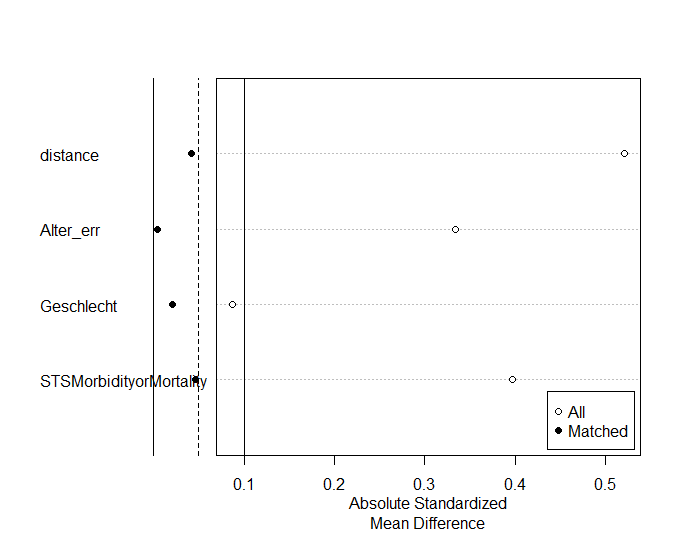
**

| **Summary of Balance for Matched Data** | MeansTreated | Means Control | Std. Mean Diff. | Var. Ratio | eCDF Mean | eCDF Max | Std. Pair Dist. |
| --- | --- | --- | --- | --- | --- | --- | --- |
| distance | 0.2819 | 0.2753 | 0.042 | 1.2387 | 0.0022 | 0.0314 | 0.0435 |
| Age | 79.6492 | 79.6806 | -0.0048 | 1.0536 | 0.0136 | 0.0524 | 0.9795 |
| Sex | 1.5131 | 1.5236 | -0.0209 | 1.0015 | 0.0052 | 0.0105 | 0.9612 |
| STS risk score | 24.5202 | 24.1264 | 0.0458 | 0.9943 | 0.028 | 0.0785 | 0.8399 |
| **Percent Balance Improvement** | Std. Mean Diff. | Var. Ratio | eCDF Mean | eCDF Max |  |  |  |
| distance | 92 | 75.6 | 98.8 | 90 |  |  |  |
| Age | 98.6 | 85.9 | 75.9 | 63.1 |  |  |  |
| Sex | 76.1 | 90.5 | 76.1 | 76.1 |  |  |  |
| STS risk score | 88.5 | 98.9 | 78.1 | 64.1 |  |  |  |

Supplemental Figure 2: Standardized mean differences for the propensity score matched population.


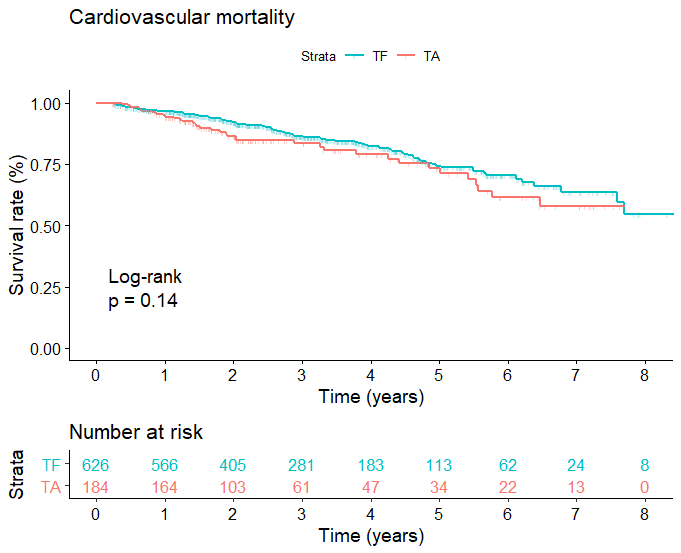

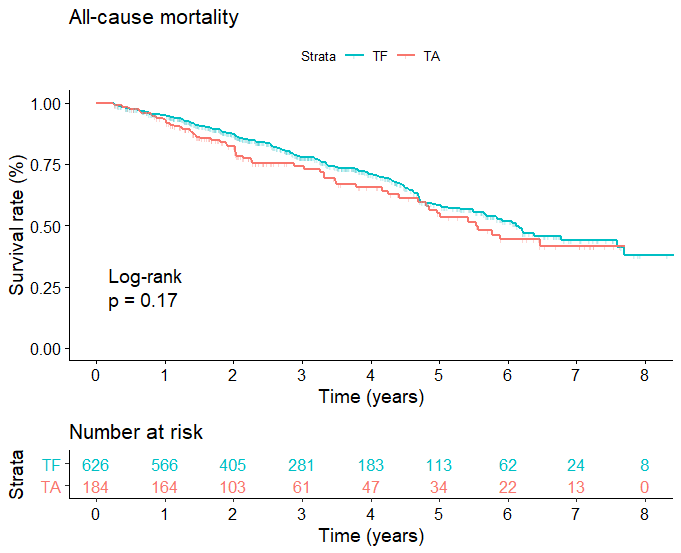


Supplemental Figure 3: Kaplan-Meier curves with accompanying risk table of (A) cardiovascular and (B) all-cause mortality of the full population, starting at 90 days post-intervention.


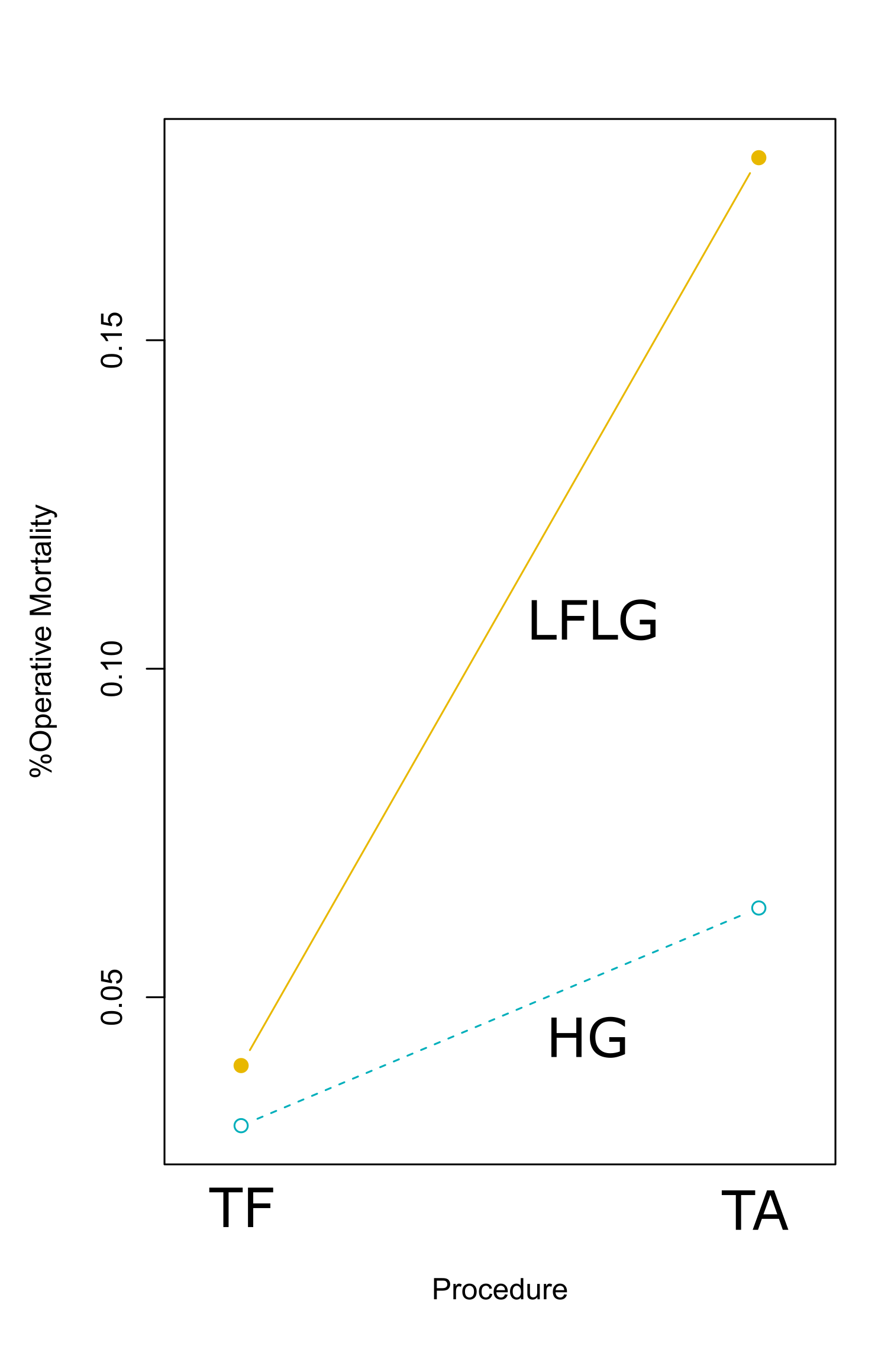


Supplemental Figure 4: Comparison of operative moratlity rates between TA and TF TAVR, startified by HG and LFLG AS.

**Supplemental Tables**

| **Variable** | **All (382)** | **TF (191)** | **TA (191)** | **p-value** |
| --- | --- | --- | --- | --- |
| **Age** | 81.00 (76.00-84.00) | 81.00 (76.00-84.00) | 81.00 (76.00-84.00) | 0.96 |
| **Female** | 198 (51.83%) | 100 (52.36%) | 98 (51.31%) | 0.16 |
| **BMI (kg/m^2^)** | 25.81 (23.15-29.02) | 25.83 (23.04-29.20) | 25.78 (23.44-28.72) | 0.76 |
| **STS risk of morbidity or mortality (%)** | 22.66 (18.35-28.78) | 22.19 (18.29-28.58) | 24.52 (18.36-28.80) | 0.44 |
| **R2-CHA2DS2-VASc** | 5.00 (4.00-7.00) | 5.00(4.00-7.00) | 5.00 (4.00-6.00) | 0.93 |
| **LVEF (%)** | 55.00 (45.00-60.00) | 55.00 (45.00-61.50) | 55.00 (46.00-60.00) | 0.68 |
| **Mean syst. Gradient (mmHg)** | 48.00 (40.00-60.00) | 50.00 (40.00-60.00) | 47.00 (40.00-60.00) | 0.23 |
| **Max. syst. Gradient (mmHg)** | 80.00 (66.50-92.00) | 80.00 (68.00-92.00) | 80.00 (64.00-94.00) | 0.45 |
| **Aortic valve area** | 0.60 (0.50-0.75) | 0.60 (0.50-0.73) | 0.62 (0.50-0.76) | 0.39 |
| **eGFR** | 67.20 (41.63-82.12) | 66.82 (41.67-81.52) | 67.27 (41.80-83.03) | 0.78 |
| **Arterial hypertension** | 328 (85.86%) | 167 (87.43%) | 161 (84.29%) | 0.17 |
| **Diabetes mellitus II** | 123 (32.20%) | 64 (33.51%) | 59 (30.89%) | 0.81 |
| **CAD** | 192 (50.26%) | 95 (49.74%) | 97 (50.79%) | 0.61 |
| **Apoplex/TIA** | 57 (14.92%) | 25 (13.09%) | 32 (16.75%) | 0.89 |
|  |  |  |  |  |

Supplemental Table 1: Baseline characteristics of the propensity score-matched subgroup.

| **Multiple regression** | **Adjusted Hazard ratio** | **95%-CI** | **p-value** |
| --- | --- | --- | --- |
| **STS risk score** | 1.07 | 0.90-1.23 | 0.41 |
| **LFLG** | 3.37 | 1.37-8.02 | **0.006** |
| **Creatine** | 1.14 | 0.72-1.62 | 0.53 |
| **Procedure (TA)** | 3.03 | 1.22-8.59 | **0.02** |

Supplemental Table 2: Multiple binomial regression analysis of operative mortality as response in the propensity score-matched cohort. Regressor selection was based on univariate regression analyses shown in the main text. Periprocedural complications were omitted due to their obvious influence on mortality in order not to overfit the model.

|  | **All (146, 16.04%)** | **Reduced LVEF (n=87, 59.59%)** | **Preserved LVEF (n=59, 40.41%)** | **p-value** |
| --- | --- | --- | --- | --- |
| **All-cause mortality** | 50 (34.25 %) | 36 (41.38%) | 14 (23.73%) | **0.03** |
| **Cardiovascular mortality** | 32 (21.92 %) | 24 (27.59%) | 8 (13.56%) | **0.04** |
| **Operative mortality** | 12 (8.22 %) | 8 (9.20%) | 4 (6.78%) | 0.60 |

Supplemental Table 3: Mortality outcomes compared between LFLG AS patients with reduced or preserved LVEF.
